# The Value of Internal Medication in Diseases of the Skin

**Published:** 1907-10-26

**Authors:** 


### Dermatology.

### THE VALUE OF INTERNAL MEDICATION IN DISEASES OF THE SKIN.

A distinguished modern dermatologist has said that "dermatology is a branch of medicine of which visibility is the prominent characteristic." This, being freely admitted, should furnish an additional plea, if such be necessary, for the systematic constitutional treatment of skin diseases. The average person sees a rash upon his skin and, not unnaturally, ascribes its presence to some disordered state of the blood. In hospital and dispensary practice patients still believe that they have not been fully dealt with unless they have been given "bottle-medicine" for their skin eruptions in addition to external remedies. Among the more cultured classes the same belief holds sway, though in a modified form, and who shall say that there is not some reason for it?

Putting aside purely parasitic affections, it may be safely said that there are few, if any, cutaneous eruptions which may not be benefited by internal treatment, and this apart from accidental sytemic disorders. Indeed, in parasitic cases, too, we have often to think very seriously of giving medicine to improve the condition of the blood, or, in other words, to increase the resisting powers of the body against hostile organisms. The older dermatologists, represented by Willan and Bateman, were accustomed to give iron in cases of ringworm of the scalp, believing that it attacked children "of feeble and flabby habit." We are in danger, nowadays, of overlooking the soil factor in parasitic diseases, and consequently the attention is focussed almost exclusively upon local treatment. The proper use of sulphur in scabies is, of course, external, but in how many cases has not the patient swallowed "brimstone and treacle" or taken "sulphur tablets" before seeking medical advice? Sometimes, however, an eczema or a dermatitis from an irritant resembles scabies very closely, in which case the use of a mild laxative internally may be most beneficial. Auto-intoxication resulting from chronic constipation leaves its mark upon the skin, and the effect of a saline purge upon such disorders as acne, common urticaria, eczema, and many of the erythemata, is often little short of miraculous.

Much has been written about the use and abuse of arsenic in diseases of the skin. At one time this drug was regarded as nothing less than a panacea for most cutaneous disorders, but nowadays it is less often administered. In lichen planus, pemphigus, and one or two of the rarer diseases, it certainly does well, and it would seem, for these affections, to possess almost a specific action. Arsenic is contra-indicated in acute inflammatory conditions, and if given in these it tends to aggravate them and sometimes to induce vesicular or bullous complications. In eczema of the nails, and in some conditions of chronic induration of the skin arsenic may be of some value.

Thyroid extract has been credited with producing good results in alopecia areata and psoriasis, and there are cases in which it has acted like a charm. Sometimes it is very disappointing, and at all times a watch must be kept upon the circulation during its administration. At the first sign of giddiness or rapidity of the pulse the drug should be stopped altogether.

The value of ichthyol, or of its newer ally, thigenol, is well seen in rosacea, acne, and urticaria, where its tonic vaso-motor action appears to good effect. Many erythematous, urticarial, and purpuric eruptions are greatly benefited by the exhibition of those drugs which are known to exercise a powerful influence upon the blood-vessels. Thus, minute doses of ergot are sometimes helpful in common urticaria, and the same remedy has recently been shown by Savill to be of real value in that rare affection known as *epidermolysis bullosa hereditaria*. Calcium chloride, again, by increasing the coagulibility of the blood, is of signal service in the above-mentioned group of skin diseases.

Iodide of calcium has lately been recommended for the treatment of ulcers of the leg, in two or three grain doses, three times a day. In some cases it certainly seems to hasten matters, but appropriate local treatment cannot, of course, be dispensed with at the same time. The effects of potassium iodide, like those of mercury, are well known.

Enough has been said to indicate the importance of internal medication in diseases of the skin, for it is only by a thorough examination of the other systems of the body as well as the cutaneous that we can hope to do the best for our skin patient.

